# The prediction of visual stimuli influences auditory loudness discrimination

**DOI:** 10.1007/s00221-014-4001-2

**Published:** 2014-07-01

**Authors:** Andrea Desantis, Pascal Mamassian, Matteo Lisi, Florian Waszak

**Affiliations:** 1 Université Paris Descartes, Sorbonne Paris Cité, Paris, France; 2 CNRS (Laboratoire Psychologie de la Perception, UMR 8242), Paris, France; 3 Institute of Cognitive Neuroscience, University College London, London, UK

**Keywords:** Crossmodal perception, Audio–visual interaction, Prediction error, Action prediction, Voluntary action control

## Abstract

The brain combines information from different senses to improve performance on perceptual tasks. For instance, auditory processing is enhanced by the mere fact that a visual input is processed simultaneously. However, the sensory processing of one modality is itself subject to diverse influences. Namely, perceptual processing depends on the degree to which a stimulus is predicted. The present study investigated the extent to which the influence of one processing pathway on another pathway depends on whether or not the stimulation in this pathway is predicted. We used an action–effect paradigm to vary the match between incoming and predicted visual stimulation. Participants triggered a bimodal stimulus composed of a Gabor and a tone. The Gabor was either congruent or incongruent compared to an action–effect association that participants learned in an acquisition phase.We tested the influence of action–effect congruency on the loudness perception of the tone. We observed that an incongruent–task-irrelevant Gabor stimulus increases participant’s sensitivity to loudness discrimination. An identical result was obtained for a second condition in which the visual stimulus was predicted by a cue instead of an action. Our results suggest that prediction error is a driving factor of the crossmodal interplay between vision and audition.

## Introduction

Traditionally, research on perception has studied each sensory modality in isolation. However, more recently, the interdependency between different senses has become a major focus of interest. This research indicates that the interplay between vision, touch and audition might be rather tight and begins at a surprisingly early level (for a review see Driver and Noesselt 2008; Alais et al. 2010). One critical factor for crossmodal interaction is the co-occurrence in time of different sensory stimulations. A number of studies using different neurophysiological methods demonstrates that brain regions traditionally labeled unimodal sensory areas are influenced by simultaneous multisensory stimulation (Senkowski et al. 2005; Lakatos et al. 2007; Kayser and Logothetis 2007). This is even the case when the different modalities do not simply provide independent samples about the same external property, but when the co-stimulation in one modality does not convey any information about the target in another modality. For example, Lakatos et al. (2007) showed that tactile stimuli modulate early responses to auditory stimuli in the primary auditory cortex of macaques. Moreover, psychophysical experiments have shown that detection judgments concerning the visual modality can be enhanced when a sound co-occurs at the location of the visual event to be detected (e.g., McDonald et al. 2000), although a maximum benefit is not necessarily obtained when there is perfect synchrony (Otto et al. 2013). Spence and Driver (2004) demonstrated that judgments of visual color can be improved by nearby touch. More recently, Kim et al. (2012) showed that auditory motion stimuli improve performance in a concurrent visual motion-detection task, even when they do not provide any useful information for the task.

In the present study, the modulatory modality does not convey any task-relevant stimulus information. It is important not to confuse this type of crossmodal interplay with multisensory integration as classically studied in paradigms where information about the same stimulus is provided by two or more different senses. These studies investigate how the perceptual system uses combined information from two or more modalities, weighting each modality’s contribution by its reliability, to yield a joint estimate of the distal stimulus. This weighted combination of individual estimates is a way to obtain an optimized joint estimate. In these cases, multisensory effects are often considered to reflect the statistical advantage of the combined use of information from the separate modalities (Alais and Burr 2004; Ernst and Bülthoff 2004; Driver and Noesselt 2008).

All of the studies outlined above indicate that different processing pathways interact, apparently even at early processing levels. Processing in, say, the auditory system is altered by the mere fact that, say, visual input is processed at the same time. However, note that processing in the modulating pathway is itself subject to diverse influences. For instance, from a predictive coding perspective, perceptual processing heavily depends on the degree to which a stimulus is predicted (e.g., Friston 2005). In this framework, each level in the processing pathway feeds back predictions about the probable input to the next lower level. Here, prediction and input are compared against each other. Mismatches between predicted and observed input are fed forward to the next level in the hierarchy, where, in turn, the predictions are adjusted so as to eliminate prediction error at the lower level. Hence, only unpredicted stimuli yield a large prediction error and adjustment response. The question, thus, arises whether the modulatory influence of one processing pathway on another pathway depends on whether or not the stimulation in this pathway was predicted.

A powerful way to manipulate perceptual prediction is action. It has been suggested that voluntary actions are guided by the ideomotor principle (Lotze 1852; Harless 1861; James 1890; for a review see Shin et al. 2010; Waszak et al. 2012). Performing an action, so the theory claims, results in a bidirectional association between the action’s motor code and the sensory effects the action produces. Once acquired, these associations can be used to select an action by anticipating or internally activating their perceptual consequences (Greenwald 1970; Prinz 1997; Elsner and Hommel 2001; Herwig et al. 2007). The ideomotor principle has been corroborated by a number of studies (Hommel et al. 2001; Schütz-Bosbach and Prinz 2007; Shin et al. 2010; Waszak et al. 2012). For example, it has been shown that participants are less sensitive to perceptual events predicted by their own actions compared to the same events that are not predicted by their action (Cardoso-Leite et al. 2010; Hughes et al. 2013; Roussel et al. 2013). Roussel et al. (2013) explain this phenomenon in terms very similar to predictive coding. In their model, the neural response to incoming stimulation is smaller because the action triggers pre-activation in the sensory areas coding for the predicted effect.

In the present study, we use an action–effect paradigm to vary the match between incoming and predicted visual stimulation (cf. Cardoso-Leite et al. 2010; Elsner and Hommel 2001; Roussel et al. 2013). We then investigate whether the match/mismatch between the prediction of a visual stimulus and the true stimulus modulates the perception of a concomitant auditory effect that was in no way related to the visual stimulus. To be more precise, participants trigger a bimodal stimulus composed of a visual Gabor patch and an auditory pure tone. The Gabor patch can either be congruent or incongruent compared to an action–effect association that participants learned in a previous acquisition phase. We test the influence of action–effect congruency on the perception of the loudness of the tone. In addition, we compare the action condition with a condition in which the visual stimulus is predictable by a cue instead of an action in order to assess any difference between motor predictive systems and more general predictive processes. We observed that incongruent–task-irrelevant Gabor patches increase participants’ sensitivity to loudness discrimination. The same held true for a second condition in which the visual stimulus was predicted by a cue instead of an action. The implication of these results is considered in more detail in the “Discussion” section.

## Methods

### Participants

Seventeen subjects (average age = 26.46 years, SD = 5.72 years) participated in the experiment for an allowance of € 10/h. All had normal or corrected-to-normal vision and hearing and were naïve as to the hypothesis under investigation. They all gave written informed consent.

## Material

Stimulus presentation and data acquisition were conducted using the psychophysics Toolbox (Brainard 1997; Pelli 1997) for Matlab 7.5.0 running on a PC connected to a 19-in. 85 Hz CRT monitor. Auditory stimuli were presented via a pair of headphones.

### Stimuli and procedure

Participants completed two blocks: an *action*-*to*-*stimulus* and a *cue*-*to*-*stimulus* block. Within each block, participants completed ten acquisition phases and ten test phases in an ABAB order. Block presentation was counterbalanced across subjects. Participants completed the experiment in two sessions.

#### Acquisition phases

**Fig. 1:**
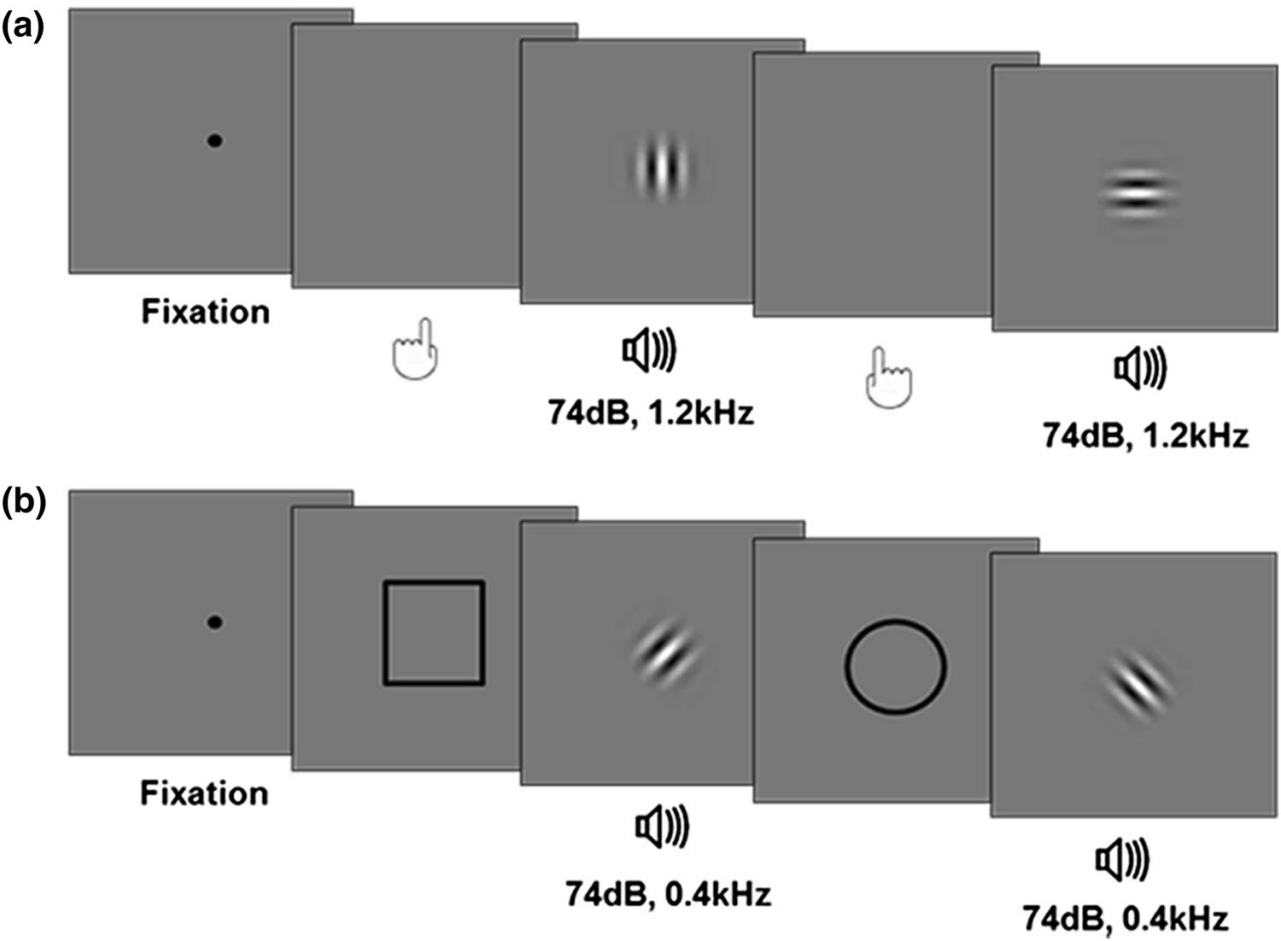
Schematic illustration of the acquisition phase. **a** In the action-to-stimulus condition, participants’ actions were followed by a bimodal stimulus composed of a pure tone and a Gabor patch. Each action generated the same tone (e.g., 1,200 Hz tone at 74 dB) and a different Gabor (e.g., left action—vertical Gabor; right action—horizontal Gabor). **b** In the cue-to-stimulus condition, participants’ were asked to remain passive and bimodal stimuli were preceded by one of two visual cues (*circle of square*). As in the action block, each one of the two cues was followed by the same tone (e.g., 400 Hz tone at 74 dB) but by a different Gabor (e.g., the *circle* was followed by a left-tilted Gabor, and the *square* by the right-tilted Gabor)

The aim of the acquisition phases was to build action–stimulus and cue–stimulus associations. In the *action*-*to*-*stimulus* acquisition phases, participants were required to execute sequences of left and right key-presses in a random order. They were asked to execute left and right actions about equally often. Feedback on the proportion of right and left key-presses was provided every 20 trials. Each action generated a bimodal stimulus composed of a pure tone and a Gabor patch presented simultaneously. The bimodal stimulus was presented for 200 ms with a stimulus onset asynchrony (SOA) of 200 ms. To be precise, each key-press triggered the occurrence of the same tone, i.e., 1,200 Hz high tone (or 400 Hz tone, depending of the group participants were assigned to; see below) at 74 dB, but of a different Gabor patch, i.e., left action—vertical Gabor (or left-tilted Gabor, see below), right action—horizontal Gabor (or right-tilted Gabor, see below). Action–Gabor mappings were counterbalanced across subjects. The Gabor patches used in the experiment had the following properties: stimulus size SD = 0.86°, spatial frequency of 2 cycles/deg. Visual stimuli were viewed from a distance of about 60 cm (see Fig. 1a for a schematic representation of the acquisition phase).

In the *cue*-*to*-*stimulus* acquisition phase, participants were required to remain passive (see Fig. 1b). In this condition, bimodal stimuli, instead of being generated by participants’ actions, were preceded by one of two possible visual cues: an empty circle and an empty square (cues size, 7.5° of width and 0.15° of thickness). Visual cues were presented in a random order and equally often. Their duration was 200 ms. Since in the action-to-stimulus condition participants had to monitor the amount of left and right key-presses, in an attempt to make the action-to-stimulus and the cue-to-stimulus conditions as similar as possible, we asked the participants to monitor the number of circles and squares presented and to indicate whether they had been presented approximately the same number of times. The onset of the sensory cues was individually yoked to the movement production times recorded in the action acquisition phase. However, if the participant started the experiment with the *cue*-*to*-*stimulus* condition, the onset of the cues was yoked to the action production times of the previous participant. Note that, for one and the same participant, bimodal stimuli presented in the *cue*-*to*-*stimulus* blocks were different from those presented in the *action*-*to*-*stimulus* blocks. For half of the participants in the action-to-stimulus condition, the stimuli were 1,200 Hz tones combined with vertical and horizontal Gabor patches. For these subjects, in the cue-to-stimulus condition, bimodal stimuli were composed of 400 Hz tones and left- and right-tilted Gabor patches. For the other half of the participants, the reversed combination of tone and Gabor patches was used: In the action-to-stimulus condition, we used 400 Hz tones combined with left- and right-tilted Gabor patches, and in the cue-to-stimulus condition bimodal stimuli were composed of 1,200 Hz tones and vertical and horizontal Gabor patches.

Each acquisition phase consisted of 80 trials, except for the first acquisition phase of 150 trials. Fourteen percent of all trials were catch trials, where participants had to indicate the orientation of the Gabor patch presented.

#### Test phases

As for the acquisition phases, in the *action*-*to*-*stimulus* and in *the cue*-*to*-*stimulus* test phases, bimodal stimuli were preceded by participants’ actions or by one of two visual cues, respectively. 800–1,200 ms after the presentation of the bimodal stimulus, a second bimodal stimulus with the same identity was presented (same tone frequency and same Gabor patch orientation). At the end of each trial, participants were required to compare the loudness of the tone (74 dB) of the standard (first) bimodal stimulus with the tone of the comparison (second) bimodal stimulus (Fig. 1). The comparison tone was of the same frequency and duration as the standard tone but varied in magnitude. Its magnitude level varied randomly between 71 and 77 dB in 1 dB steps. The participants judged which of the two tones (the standard tone or comparison tone) was louder by pressing with their feet one of two response buttons.

**Fig. 2:**
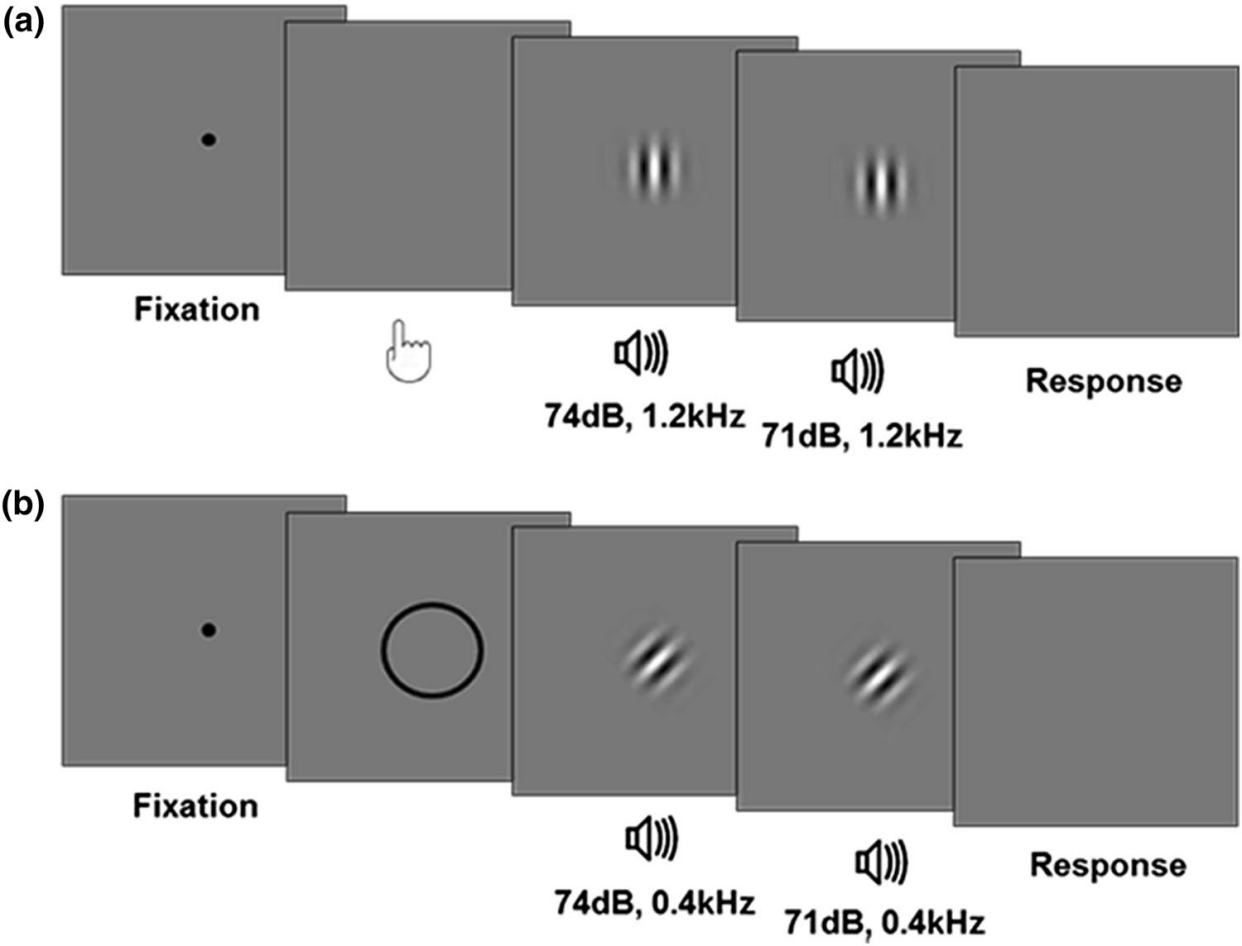
Schematic illustration of a test trial. **a** In the action-to-stimulus condition, participants’ action triggers a first bimodal stimulus. The Gabor patch can either be congruent or incongruent with respect to the action–effect association participants learned in the previous acquisition phase. After a variable delay of 800–1,200 ms, a second bimodal stimulus is presented. Participants indicate whether the first or second tone was louder. **b** The cue-to-stimulus condition is identical to the action condition except for the fact that participants’ actions are replaced by visual cues

In both the action and the cue-to-stimulus conditions, participants completed congruent and incongruent trials in which the associations they learned in the previous acquisition phases between left/right action (*action*-*to*-*stimulus* condition) and circle/square shape (*cue*-*to*-*stimulus* condition), and the visual modality of the bimodal stimulus was respected or violated, respectively. For instance, if in the *action*-*to*-*stimulus* acquisition phase, the left key-press triggered a high pitch tone and a vertical Gabor, in half of the trials of the test phase the same action triggered the same pitch and the same Gabor orientation (congruent trials), and in the other half the action triggered the same pitch but the orientation that was associated with the other action (incongruent trials). Incongruent trials were randomly distributed between the fourth and the last trial of each test phase, in order to strengthen the association between action and effect (Fig. 2).

The action and cue-to-stimulus test phases consisted of 320 trials for a total of 640 trials [160 × 2 (congruent and incongruent conditions) × 2 (action and cue-to-stimulus conditions)]. For both the action and the cue-to-stimulus conditions, the test phase was completed in 10 short mini-blocks of 32 trials each.For both congruency conditions, each of the seven comparison tone magnitudes was made 20 times (for a total of 140 × 2 congruency condition). The rest of the trials (20 × 2 congruency conditions) were catch trials where participants were required to indicate the orientation of the Gabor patch that followed their action or the cue. Note that in these trials, participants were not required to compare the loudness of the tones since no comparison stimulus was presented. Catch trials were randomly distributed. Participants were unaware at the beginning of each trial whether or not the trial was a catch trial.

### Data analysis

**Fig. 3:**
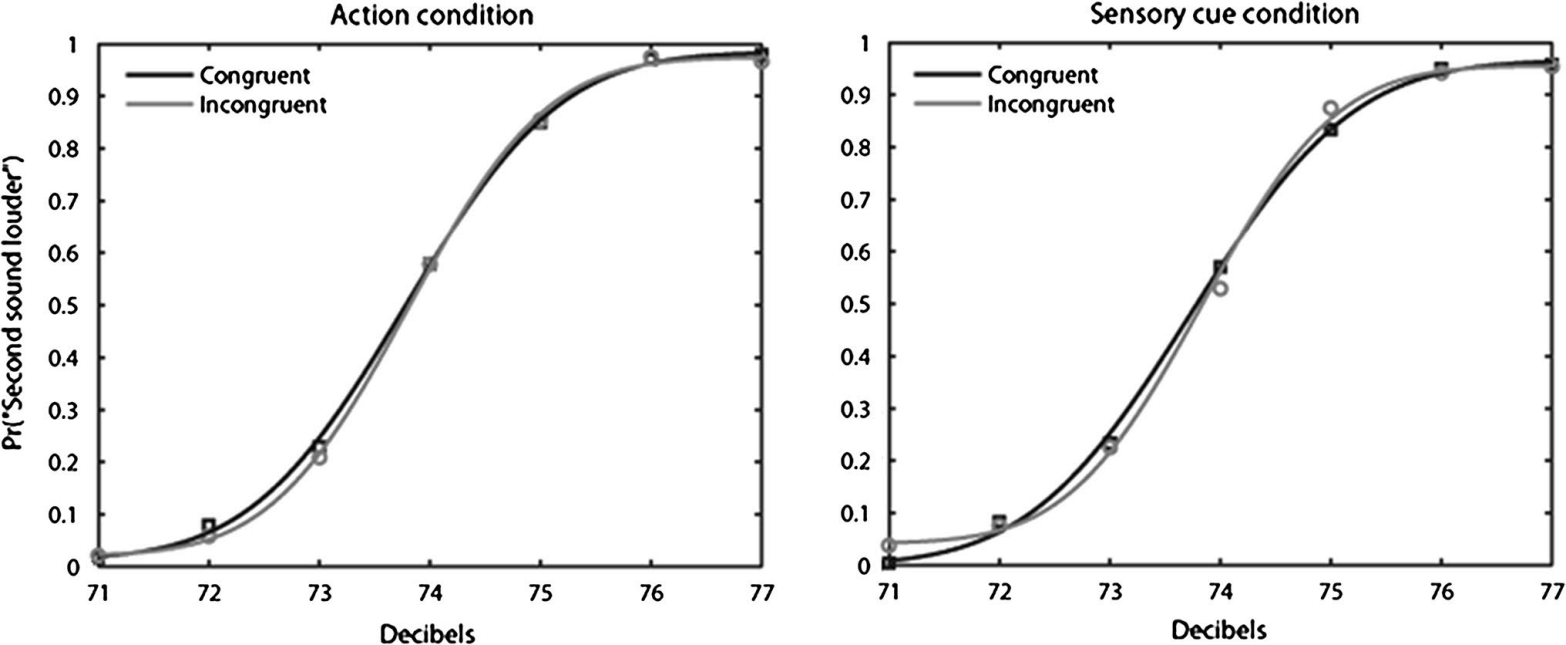
Proportion of “second tone louder” responses for the action congruent/incongruent and sensory cue congruent/incongruent conditions (averaged across all participants) as a function of the seven comparison tone magnitudes

The proportion of “second tone louder” responses was calculated separately for each participant, condition and the seven magnitudes of the comparison tone. Psychometric functions (cumulative Gaussians) were fitted using the Psignifit Toolbox version 2.5.6 for Matlab (see http://bootstrap-software.org/psignifit/) which implements the maximum likelihood method described by Wichmann and Hill (2001) (see Fig. 3). Based on each individual function, we calculated the point of subjective equality (PSE) and the just noticeable difference (JND). The PSE, defined as the comparison tone magnitude judged as louder than the first tone on 50 % of trials, reflects the perceived intensity of the first tone under the different conditions. Lower PSE values in one condition would indicate a reduction of the perceived intensity of the standard tone in that condition compared to the other. However, it should be noted that the PSE could be tainted by various response biases, which makes its interpretation difficult. For instance, PSE can be affected by a change in decision criterion (i.e., preference of saying that congruent stimuli are less loud than incongruent) and not by a sensitivity change. The JND, defined as half the difference of the comparison tone magnitude judged as louder on 75 % and on 25 % of trials, is a measure of the slope of the psychometric function. As such, it reflects participants’ sensitivity to loudness discrimination. Large JND values reflect poor sensitivity. The level of significance of our analysis was set at *p* < .05 for all statistical tests. Two participants were excluded from the analyses due to the extremely poor performances on catch trials (correct responses <50 %).

## Results

To investigate whether the conditions varied in terms of allocation of attentional resources, we conducted a repeated measures analysis of variance (ANOVA) on catch trials performances with causality (action-to-stimulus and cue-to-stimulus) and congruency (congruent and incongruent) as factors. The analysis of catch trials showed no main effect of causality *F*(1, 14) = .857, *p* = .370, no main effect of congruency *F*(1, 14) = 3.237, *p* = .093 and no significant interaction between causality and congruency *F*(1,14) = 0.57, *p* = .815 (action congruent: *M* = 94.67, SD = 7.43; action incongruent: *M* = 91.67, SD = 7.94; cue congruent: *M* = 96.33, SD = 3.99; cue incongruent *M* = 92.67, SD = 7.76), suggesting that attentional processes are minimally affected by our manipulations.

**Fig. 4:**
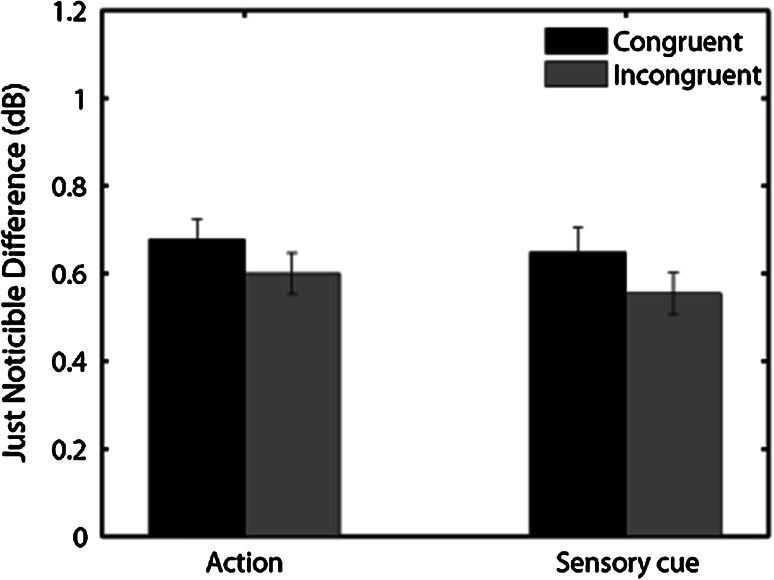
Mean JND values (in dB) per congruency and action/cue-to-stimulus conditions. *Bars* represent standard errors

We, then, conducted two repeated measures analyses of variance (ANOVA) on PSE and JDN values, respectively, with causality and congruency as factors. The analysis of the PSE values showed no main effect of causality, *F*(1, 14) = .357, *p* = .559, no main effect of congruency, *F*(1, 14) = .105, *p* = .749 and no interaction, *F*(1, 14) = .004, *p* = .949. In contrast, the analysis of JND values revealed a main effect of congruency, *F*(1, 14) = 5.288, *p* = .037, with higher JND values in the congruent versus incongruent trials (see Fig. 4), showing, thus, a reduction of sensitivity in congruent compared to incongruent trials. We did not observe any effect of causality *F*(1, 14) = .912, *p* = .355, nor an interaction *F*(1, 14) = .029, *p* = .865.

Mean *r* ^2^—as a measure of the goodness of fit for the four conditions were as follows: action congruent *M* = 0.9842, SD = 0.0124; action incongruent *M* = 0.984, SD = 0.0133; sensory cue congruent *M* = 0.9853, SD = 0.0150; and sensory cue incongruent *M* = 0.9819, SD = 0.0216.

## Discussion

In the present study, we observed that unpredicted task-irrelevant visual stimuli enhance loudness discrimination compared to predicted visual stimuli, both when these stimuli are self-generated and when they are predicted by a cue. The current findings, thus, clearly show that crossmodal influences from the visual to the auditory domain depend on whether or not the modulatory visual stimulus was predicted.

Concerning the neurophysiological basis of non-informative crossmodal influences as described in the introduction and as demonstrated in the present study, Driver and Noesselt (2008) distinguish three (mutually not exclusive) accounts. First, there might be direct cortico-cortical routes between the different primary sensory areas, as reported, for example, between auditory areas and primary visual cortex in the macaque brain (Falchier et al. 2002). Second, multisensory convergence zones exist at earlier levels than traditionally assumed. Third, multisensory influences on unimodal sensory cortex could be based on neural feedback from multisensory areas (Macaluso et al. 2000). These accounts, or a combination of them, can explain multisensory enhancement of brain regions that are traditionally labeled unimodal sensory areas, extremely early multisensory ERP modulations, and also—as demonstrated in the present study—multisensory influences on perceptual indices (Senkowski et al. 2005; Lakatos et al. 2007; Kayser and Logothetis 2007; Kim et al. 2012).

The current research is not meant to differentiate between the three accounts. However, independent of the specific mechanisms the effects reported above are based on, the current findings suggest that the signal driving the modulation has to be understood in terms of predictive coding. Namely, it seems that the driving factor of the crossmodal interplay between vision and audition is, at least partially, the prediction error of the comparison between prediction and input. Several scenarios are possible.

The simplest account for the current findings would be to assume that direct cortico-cortical connections between visual and auditory areas receive input from error units in the visual cortex, such that processing in the auditory cortex is facilitated as a function of the prediction error. For instance, a prediction error in the visual cortex would lead to an enhancement of the processing in the auditory stimulus, thus resulting in better discrimination in the incongruent compared to the congruent condition. Accordingly, one would predict that larger errors result in more crossmodal influence.

Another interesting explanation is based on the notion of audiovisual integration. It is conceivable that a stable representation of an audiovisual object is created by learning a particular audiovisual combination in the acquisition phase. The representation of this audiovisual object is then reactivated in the test phase only in the congruent trials. Accordingly, associative areas that represent the audiovisual object would influence the processing of unimodal areas. For instance, the detection by multisensory/associative areas of an audiovisual incongruence would feedback onto unimodal regions increasing the processing of the new stimulation, thus increasing participants sensitivity to incongruent audiovisual stimuli (cf., Odgaard et al. 2004).

Please note that, as Driver and Noesselt (2008) argue, effects based on the mere co-stimulation in another modality appear to reflect rather nonspecific influences related to rapid alerting, arousal, or the weighting of modalities. An event in one modality might facilitate processing in another modality in that it makes a stimulus salient in space and/or time Alternatively, processing two modalities simultaneously might induce a global cost, for instance in processing time (Otto and Mamassian 2012). Accordingly, in our experiment, incongruent stimuli might have increased alertness of the system in the presence of an unusual event, thus facilitating processing of the auditory stimulus. Note that, irrespective of witnessing a gain or a cost, the notion of unspecificity does not make this interplay between the senses less genuine (Driver and Noesselt 2008). Moreover, this type of unspecific mechanism makes sense, as stimulus analysis becomes more important if a perceptual prediction turned out to be wrong. This mechanism may warrant that flawed perceptual inference in one modality is supported by inference in another modality, or that surprising events in the environment are more deeply processed to backup the new interpretation of the environment.

Another interesting aspect of our results is the absence of a difference in PSE and JND between prediction that results from choosing between actions (motor identity prediction) and prediction that results from predictive cues (non-motor identity prediction; cf., Hughes et al. 2013). This is in contrast with previous studies showing a decrease in sensitivity for stimuli that are predicted from an action compared to those predicted by an external cue (Cardoso-Leite et al. 2010). An important aspect of our experiment that might explain the absence of a difference between action and sensory cue trials in the present study could be the fact that in our experiment predictive cues were presented on the screen for 800–1,000 ms. This might have provided enough time to the participant to integrate efficiently the predictive value of the cue and, in turn, to learn cue–stimulus associations efficiently. Moreover, note that recent studies have reported sensory attenuation for stimuli that are externally generated, suggesting that the predictive mechanisms involved in the phenomenon are not limited to action prediction (Lange 2009; Vroomen and Stekelenburg 2009). Predictive mechanisms have also been invoked to understand another type of attenuation, viz. repetition suppression (Summerfield et al. 2008). These studies suggest that prediction is strongly utilized outside the motor system. Interestingly in this context, recent studies suggest the prediction of external events partly involves our motor system and in particular the pre-motor cortex (Schubotz and von Cramon 2002; Schubotz 2007; Bubic et al. 2010). Finally, we did not observe any difference of PSEs between congruent and incongruent conditions. This might suggest that identity prediction instead of inducing a response bias alters the processing of the sensory signal as it has been suggested by recent studies (cf. Cardoso-Leite et al. 2010; Roussel et al. 2013).

To summarize, the current findings clearly indicate that crossmodal influences from the visual to the auditory domain depend, at least partially, on whether or not the modulatory visual stimulus was predicted.
